# Keratin 5 expression in squamocellular carcinoma of the head and neck

**DOI:** 10.3892/ol.2014.2591

**Published:** 2014-10-09

**Authors:** VIRGIL VASCA, ELISABETA VASCA, PAUL FREIMAN, DIANA MARIAN, AMALIA LUCE, MASSIMO MESOLELLA, MICHELE CARAGLIA, FILIPPO RICCIARDIELLO, TATIANA DUMINICA

**Affiliations:** 1 Faculty of Medicine, Pharmacy and Dental Medicine, ‘Vasile Goldiş’ Western University of Arad, Arad 310025, Romania; 2 Department of Biochemistry, Biophysics and General Pathology, The Second University of Naples, Naples I-80138, Italy; 3 Department of Otolaryngology, Head and Neck Surgery, The University of Naples ‘Federico II’, Naples I-80131, Italy

**Keywords:** squamocellular carcinoma, cytokeratins, tumors, immunohistochemistry, markers

## Abstract

Keratin 5 (K5) is present in the basal layer of a stratified squamous keratinized and non-keratinized epithelium. K5 and K14 have been demonstrated in the mucosa and tumors of the oral cavity, oropharynx, hypopharynx and larynx, and in the mitotic active basal cells of a stratified squamous epithelium. The aim of the present study was to assess K5 expression in squamocellular carcinoma with various localizations in the head and neck. A total of 13 biopsy fragments were included from patients diagnosed with squamocellular carcinoma of the larynx area (n=2), pharynx (n=2), hard palate (n=1), tongue (n=2), submandibular (n=1), lip (n=1), gingival sulcus (n=1), nasal pyramid (n=1), maxilla (n=1) and zygomatic (n=1). The immunohistochemical staining for K5 was evaluated according to the following score criteria: 0 (0% positive cells); 1 (<10% positive cells); 2 (10–30% positive cells); and 3 (>30% positive cells). K5 expression was observed in all squamocellular carcinomas included in the present study with scores between 1 and 3. For well- and moderately-differentiated histopathological types, a maximum score of 3 was recorded for all of the cases, not including the laryngeal area, which presented a score of 2. The following scores were identified in the regions of the poorly differentiated carcinomas: Jaw, 3; gingival sulcus, 2; and tongue and submandibular area, 1. These observations may aid with an improved stratification of head and neck squamocellular carcinoma, thus improving the diagnosis and treatment strategies for this type of cancer.

## Introduction

Cytokeratins (CKs) are proteins of the intermediary filaments of keratin, situated intracytoplasmically and present in the cytoskeleton of all epithelial cells. The term CK was initially used at the end of the 1970s (1) when the protein subunits of the intermediary filaments from the inside of cells were identified and characterized for the first time. A novel nomenclature for keratins was established in 2006, and the proteins that were previously known as CKs were termed keratins (Ks) (2). There are two types of Ks: Acidic (type I) and basic (type II). K5 is present at the level of the basal layer of the keratinized and non-keratinized stratified squamous epithelia. K5 expression is decreased at the level of the spinosum stratum of the normal oral mucosa, and in the dysplastic epithelium, K5 is positive in the basal, parabasal and stratum spinosum cells. In the case of cancers, Ks serve predominantly as tumor markers for diagnostic procedures. Therefore, in the case of breast cancer, an association between the young age of patients at presentation and the basal-like subtype (characterized by the absence of the expression of estrogen, progesterone and human epidermal growth factor receptor 2 receptors, and the presence of epidermal growth factor receptor and K5 and K6 expression) has been recorded. Additionally, a higher tumor grade has been associated with a prognosis of approximately five years of disease-free-survival (3,4).

Squamocellular carcinomas, independently from their origin, are characterized by the expression of the Ks of the stratified epithelia (K5, K14 and K17) and overexpression of K6 and K16 in hyperproliferating strata (5). The combined overexpression of K5 and K14 was demonstrated in tumors of the oral cavity, in the oropharyngeal, hypopharyngeal and laryngeal areas (6,7) and in the basal actively mitotic cells of the squamous stratified epithelium (8). The expression of K5 and K14 remains high even if the malignant grading decreases (9–11).

Using these data as a stratification method, the aim of the present study was to identify the K5 expression features in the squamocellular carcinoma that were located in various regions of the oral and maxillofacial area in order to improve the diagnostic accuracy.

## Materials and methods

### Patients and specimens of squamocellular carcinoma

A total of 13 biopsy fragments were evaluated, which were obtained from patients that had been diagnosed with squamocellular carcinoma in the following areas; larynx (n=2), pharynx (n=2), hard palate (n=1), tongue (n=2), submandibular (n=1), lip (n=1), gingival sulcus (n=1), nasal pyramid (n=1), maxilla (n=1) and zygomatic (n=1). Patients provided written informed consent.

### Immunohistochemistry analysis

The biopsy fragments were placed into buffered formalin (10%) for 48 h and subsequently paraffin was added. All stages of the immunohistochemical technique were facilitated by the use of an automated immunohistochemistry instrument (Leica Bond-III; Leica Microsystems, GmbH, Wetzlar, Germany), according to the manufacturer’s instructions and using a primary monoclonal mouse antibody specific for CK5 (clone XM26, ready to use; Novocastra Laboratories Ltd., Newcastle upon Tyne, UK). Following dehydration in pure alcohol, the sections were placed in benzene to replace ethanol prior to embedding in paraffin and subsequently fitted using Canada balsam (Sigma-Aldrich, St. Louis, MO, USA). The microscopic evaluation was performed with a Nikon Eclipse E600 microscope (Nikon Corporation, Tokyo, Japan) and the images were obtained using the Laboratory Universal Computer Image Analysis G system (Laboratory Imaging Co., Prague, Czech Republic). The immunohistochemistry for K5 in the tumor cells was evaluated according to the following scores: 0 (0% CK5-positive cells), 1 (<10% CK5-positive cells), 2 (10–30% CK5-positive cells) and 3 (>30% CK5-positive cells).

## Results

### Score of CK5 expression in various squamocellular carcinoma

**Figure 1:**
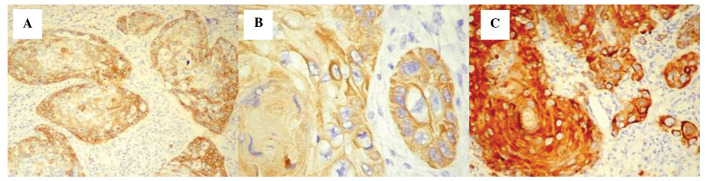
Squamocellular carcinoma. Immunohistochemical staining for cytokeratin 5 in the (A) larynx (score, 2); magnification, ×10; (B) larynx (score 3); magnification, ×40; and (C) pharynx (score, 3); magnification, ×20.

The morphological labeling indicated the presence of seven cases of well-differentiated squamocellular carcinoma (two larynx, two pharynx, one nasal pyramid, one lip and one zygomatic), two were moderately differentiated (tongue and hard palate) and four were poorly differentiated (tongue, submandibular area, maxillary and gingival sulcus). In certain cases, at the level of the larynx, scores of 2 were observed in addition to the presence of two distribution models, which were as follows: i) Homogeneous distribution, all the cells in the tumor area were positive for CK5 labeling; and ii) heterogeneous distribution, the CK5-positive cells were prevalent at the periphery of the tumor areas. However, the distribution of the immunohistochemical staining score was always 2 (Fig. 1A).

For the second case that originated from the larynx, the score was 3, the distribution was homogeneous and a prevalent cytoplasmic pattern was demonstrated (Fig. 1B).

**Figure 2:**
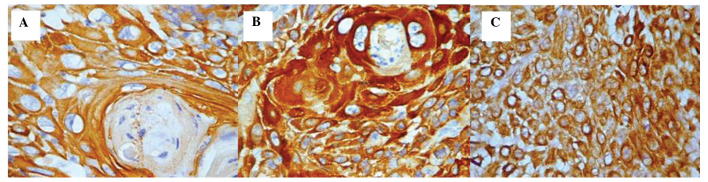
Squamocellular carcinoma. Immunohistochemical staining for cytokeratin 5 in the (A) lip (score, 3); magnification, ×40; (B) nasal pyramid (score, 3); magnification, ×40; and (C) zygomatic (score, 3); magnification, ×20.

The well-differentiated squamocellular carcinoma cases, originating from the pharynx, exhibited a CK5 expression score of 3 with a homogeneous distribution pattern and intense immunohistochemical staining (Fig. 1C). All of the squamocellular carcinoma cases originating from the lip, nasal pyramid and zygomatic area had a well-differentiated grade. These cases were all highly positive for CK5 expression (score, 3) with either a cytoplasmic or mixed (cytoplasmic and membrane) pattern and a homogeneous distribution (Fig. 2A–C).

Moderately-differentiated squamocellular carcinoma cases originating from the lip and hard palate demonstrated a homogeneous distribution of CK5 in all the cells of the tumor area with a score of 3.

### Intensity and expression pattern of CK5

**Figure 3:**
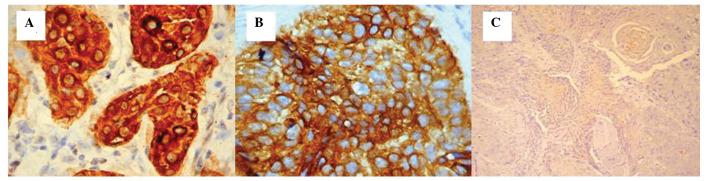
Squamocellular carcinoma. Immunohistochemical staining for cytokeratin 5 in the (A) lip (score, 3); magnification, ×40; (B) hard palate (score, 3); magnification, ×40; and (C) tongue (score, 1); magnification, ×10.

With regard to the intensity of the immunohistochemical staining and the associated expression pattern, the following were observed: i) Maximal intensity and a cytoplasmic pattern in the isolated cells, in addition to a mixed pattern in the carcinomas located in the lip (Fig. 3A). ii) Heterogeneity of the intensity of the immunohistochemical staining (moderate and intense), however, with an expression pattern that was comparable between those that originated from the lip and the carcinomas that originated from the hard palate (Fig. 3B).

For all the four cases that were included in the poorly-differentiated grade carcinoma category located in the tongue, submandibular area, maxillary and gingival sulcus, a marked reduction in the scores for the CK5 immunohistochemistry was observed; for squamocellular carcinoma originating from the tongue, a score of 1 was observed (Fig. 3C). The cells that were positive for CK5 were dispersed among the cells that did not express CK5 and exhibited a decreased reaction intensity and cytoplasmic expression pattern.

**Figure 4:**
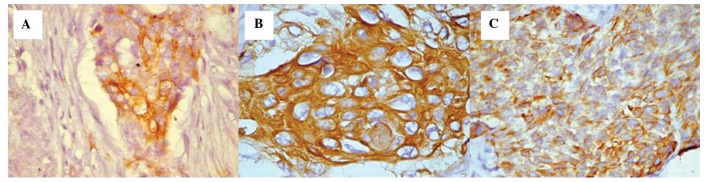
Squamocellular carcinoma. Immunohistochemical staining for cytokeratin 5 in the (A) submandibular (score, 1); magnification, ×40; (B) upper jaw (score, 3); magnification, ×40; and (C) gingival sulcus (score, 2); magnification, ×40.

The squamocellular carcinoma case originating from the submandibular area presented isolated regions with positive cells in the tumor area (score, 1) as well as cytoplasmic expression (Fig. 4A). For the case originating from the upper jaw area, a high score of 3 was observed (Fig. 4B).

**Figure 5:**
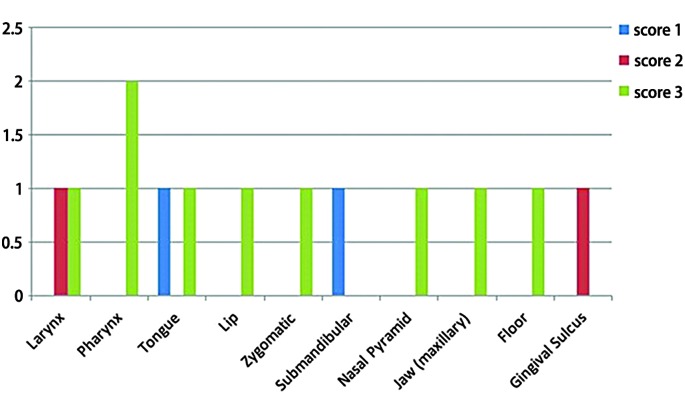
Ratio between the values of the score that indicates the percentage of the positive cytokeratin 5 tumoral cells and the various areas where the squamocellular carcinomas were located.

The squamocellular carcinoma that originated from the gingival sulcus area preserved the same mixed aspect with positive and negative cells present at the level of the tumor areas. In this case, the intensity of the reaction was moderate (score, 2) and the expression patterns were cytoplasmic or mixed (Figs. 4C and 5).

## Discussion

Expression of CK5 and CK14 was found to be specific for mucosal areas and for tumors that originated from the oral cavity, oropharynx, hypopharynx and larynx, and in the basal actively mitotic cells of the squamous stratified epithelia (8). Velluci *et al* (9) and Marley *et al* (10) observed a decreased expression of CK5 and CK14 along with malignant alterations; however, the expression did not cease completely. The presence of K5 expression was observed in all of the cases of squamocellular carcinoma that were included in the present study.

Kaufmann *et al* (12) demonstrated a high expression of CK5 and CK6 in 81% of squamocellular carcinomas that were included in their study, with an intense immunoexpression, which was diffuse in the majority of the tumoral cells. In comparison to squamocellular carcinomas, Kaufmann *et al* observed that only 14.2% of the non-squamocellular carcinomas expressed CK5 and CK6, with distribution in a reduced number of tumor cells. In the present study, all the cases of squamocellular carcinomas originating from the oral and maxillofacial areas expressed CK5 in the tumor cells, with scores ranging from 1 to 3. Whereas, the variation between the intensity scores for CK5/6 and p63 was relatively small in a previous study (12). Crook *et al* (13) highlighted that p63 expression in squamocellular carcinomas is not dependent on the tumoral grade, as it was identified to be strongly expressed in all cases of poorly-differentiated carcinoma originating from the nasopharynx. The same expression was recorded for CK5 and CK6. In the present study, a decrease of CK5 expression was observed in two of the poorly-differentiated squamocellular carcinoma cases; one originating from the tongue and the other from the submandibular area, with scores of 1 (between 10–30% of positive tumoral cells). An intermediate score of 2 was observed in the case originating from the gingival sulcus. Maintenance of the maximal score of 3 was observed in the case that originated from squamocellular carcinomas located in the upper jaw. Malzahn *et al* (14) and Tot (15) reported differences between p63, and CK5 and CK6 expression in breast carcinoma, where CK5 and CK6 were positive in 61% of the cases, compared with p63 that was expressed in 11% of the cases that were included in the study.

According to Chung *et al* (16) specific Ks, such as K14 and K15, are associated the molecular classification of squamocellular carcinomas based on gene analysis, however, the expression of CK5 and CK6 have not been analyzed. The present study promotes the introduction of CK5 in classifying the subtypes of squamocellular carcinomas.

In conclusion, CK5 was expressed in all of the types of squamocellular carcinoma that were included in the present study, with scores varying from 1 to 3 and a higher expression observed in the poorly-differentiated carcinomas as follows: A score of 3 for those originating from the jaw, 2 for the one originating from the gingival sulcus, and 1 for carcinomas of the tongue and submandibular area. The expression was present for well- and moderately-differentiated histopathological grades with a maximal score of 3 for all of the cases, with the exception of one carcinoma of the larynx where the score was 2. The present study confirms the role of CK5 in the definition of the differentiation of squamocellular carcinoma of head and neck revealing a differential expression depending on the anatomic site of the primary tumour. On these bases, additional studies on a larger series of patients are required.
